# Legitimacy, embedding, and learning framework: lessons from individual placement and support for analyzing sustainment and scale-up of complex psychosocial interventions

**DOI:** 10.3389/ijph.2026.1609505

**Published:** 2026-07-28

**Authors:** Jaakko Harkko

**Affiliations:** Faculty of Social Sciences, Helsingin Yliopisto, Helsinki, Finland

**Keywords:** evidence-based practice, implementation science, mental health services, policy implementation, supported employment

## Abstract

**Objectives:**

To develop a framework of policy-level domains for analyzing the sustainment and scale-up of Individual Placement and Support and, tentatively, other complex evidence-based psychosocial interventions.

**Methods:**

Integrative review of 41 empirical studies on contextual determinants of Individual Placement and Support (IPS) sustainment and scale-up was conducted. The results were synthesized inductively and interpreted abductively using implementation science, policy implementation research, and welfare policy literature.

**Results:**

The LEL framework organizes recurring external-context patterns into three policy-level domains: Policy Legitimacy addresses policy legitimization and decision chains linking high-level policy decisions to implementation; Policy Embedding addresses multi-level and cross-sector implementation structure; and Policy Learning addresses implementation evidence infrastructure through which implementation experience informs practice, policy, and model refinement.

**Conclusion:**

The Legitimacy–Embedding–Learning (LEL) framework proposes policy-level domains for analyzing IPS sustainment and scale-up. It functions as an integrative analytical framework that supports cumulative evidence-building and comparative research within IPS, with testable relevance for other evidence-based complex psychosocial interventions.

## Introduction

Evidence-based complex psychosocial interventions (CPIs), such as Individual Placement and Support (IPS), Housing First, and family psychoeducation, are supported by research showing improvements in social and clinical outcomes among people with mental illness [1]. CPIs specify core elements, often formalized in fidelity criteria, that are hypothesized to drive outcomes and are therefore expected to be preserved during implementation and adaptation [2]. CPIs are complex because they involve interacting practices delivered by multidisciplinary staff across organizational boundaries and must be embedded in fragmented service systems shaped by policy, institutional, funding, and implementation conditions. Hence, ambitions to expand services may require adaptations not only to the service model itself but also to the surrounding service system to enable scale-up. Accordingly, an integrative review design with an interpretive synthesis was used to (i) identify recurring external-context patterns reported in the empirical IPS implementation literature and (ii) formalize these findings into an analytic framework for knowledge-building on sustainment and scale-up.

Sustainment and scale-up depend on determinants external to individual organizations. External context includes policies and regulations, funding and reimbursement arrangements, cross-sector governance, performance management systems, labor-market conditions, and broader social norms [3–5]. While systems-oriented implementation science frameworks conceptualize implementation as a dynamic process unfolding within multi-layered and interacting contexts [6–8], empirical implementation research tends to neglect defining external context as an analytical object [9–11]. Thus, although external context is increasingly acknowledged in implementation research, the policy-level domains through which it shapes sustainment and scale-up remain insufficiently specified.

IPS offers a well-suited empirical case for framework development because it is fidelity-based, extensively studied, and implemented across mental health, social care, employment, and welfare systems. Focusing on a single well-specified intervention reduces heterogeneity and limits the risk that differences in foundational principles or delivery models confound the interpretation.

### Theoretical positioning

This article proposes an analytic framework for knowledge-building on IPS sustainment and scale-up and, tentatively, evidence-based CPIs more broadly. Existing frameworks serve different purposes: EPIS addresses phased implementation processes and contexts [12], RE-AIM/PRISM focuses on planning and evaluation across implementation outcomes and contextual influences [13], ExpandNet on scale-up strategy [14], the Dynamic Sustainability Framework on continuing intervention–context fit and perceiving sustainment as a process of continuous adaptation [15], and FRAME on intervention adaptations [16]. Schell et al.’s nine-domain Sustainability-capacity framework identifies political support, funding stability, partnerships, organizational capacity, program evaluation, adaptation, communication, public health impacts, and strategic planning as important for sustaining public health programs [17]. LEL therefore contributes at a different analytical level by asking which policy-level domains are most useful for analyzing external context’s role in sustainment and scale-up.

This article draws on policy implementation research and welfare-state scholarship as adjacent interpretive resources. They are therefore reported in the Results, where they help interpret the findings and support theory-building.

Sustainment and scale-up are discussed together because they pose related but distinct implementation challenges: sustainment as a process refers to creating conditions that allow an intervention to remain a routine, high-fidelity practice over time, and scale-up usually refers to expanding an intervention to more sites and service users under broadly similar conditions. Successful scale-up may therefore be understood as a function of the penetration and reach of sustained services. In contrast, scale-out extends an intervention to new populations and/or delivery systems. In IPS, scale-out includes youth, people with substance use disorders, and other groups whose support is organized across different service systems.

The research questions were:

- What recurring empirical patterns can be identified in the external-context conditions shaping sustainment and scale-up across empirical IPS implementation studies?
- What policy-level concepts from welfare policy analysis, policy implementation research, and implementation science help explain these patterns?
- How can these analytic constructs be integrated into a framework of policy-level domains for analyzing how external context enables or constrains sustainment and scale-up?

## Methods

### Study design

This study used a theory-building integrative review design to synthesize heterogeneous empirical evidence on external context conditions shaping IPS sustainment and scale-up. Integrative reviews aim to synthesize evidence across heterogeneous study designs and to generate conceptual insights that can inform future research and practice [18, 19]. An integrative rather than a scoping review was chosen to permit interpretive engagement with implementation science, policy implementation research, and welfare policy analysis. Empirical findings were grouped inductively and interpreted abductively to develop plausible explanations and formalize the LEL framework [20, 21].

### Sources of evidence

The empirical evidence base was built in two steps. First, empirical IPS implementation studies were drawn from a recent scoping review; 32 of the 59 reported studies were included as they reported relevant implementation outcomes: sustainment, penetration, or reach [22]. Second, the evidence base was updated through a Web of Science search from the scoping review end date through November 2025. The updated search yielded 125 records; 28 full-text articles were screened, and nine additional articles met the inclusion criteria and were added to the evidence base. Included studies are presented in Supplementary File 1. They spanned legislative contexts, welfare sectors (health, social care, and employment services), and governance levels (local, regional, and national), and used qualitative, quantitative, and mixed-method designs. No studies from low- or middle-income countries were identified; relevance in such settings therefore requires separate examination. Because the aim was conceptual synthesis, no formal quality appraisal was conducted; instead, recurring themes reported across independent studies and settings were prioritized.

Theoretical manuscripts were selected purposively for their explanatory relevance to inductively identified patterns. This approach was also appropriate because the included IPS implementation literature was not anchored in a single research paradigm and most studies addressed pragmatic implementation questions. A smaller subset engaged more explicitly with implementation structures, institutional logics, or welfare-service perspectives.

### Data extraction and analysis

Text excerpts reporting on sustainment, penetration, or reach, together with the external-context conditions discussed in relation to these outcomes were extracted. The analytical process followed Braun and Clarke’s six-phase thematic analysis [23]. The extracted material was read for familiarization, coded for external-context conditions linked to these outcomes, and grouped into candidate themes. These themes were reviewed against the extracted data and theory sources, then refined, defined, and named as broader analytical domains. The process was iterative and recursive; the final framework structure was cross-checked against empirical studies to ensure that each domain was grounded in recurring empirical patterns. This check supported the three-domain structure. The final domains were written up as an integrative framework and are presented in a summary table. Empirical examples from the included studies are provided to illustrate the domain structure.

Analyses were conducted in ATLAS.ti (version 26) for thematic coding and categorization. ChatGPT (version 5.5) and Grammarly were used for ideation and language editing/proofreading, not for data coding, analysis, interpretation, or generating results.

## Results

### Overview

The findings across the literature were grouped into three policy-level domains associated with sustainment and scale-up: Policy Legitimacy, Policy Embedding, and Policy Learning (Table 1). Policy Legitimacy concerns how policy paradigms and legitimization processes shape the political-administrative decision chains through which IPS is mandated or contested across organizations. Policy Embedding captures multi-organizational implementation structures as systems in which core implementation functions are connected or fragmented across levels and sectors. Policy Learning focuses on structures through which implementation experience informs practice, policy decisions, and model refinement. The domains are considered distinct but mutually reinforcing: legitimacy shapes whether embedding is authorized, embedding creates organizational conditions for learning, and learning can renew legitimacy and guide further embedding.

**TABLE 1:** Key features of the Legitimacy–Embedding–Learning (LEL) framework for analyzing the sustainment and scale-up (International IPS implementation literature, search through November 2025).

LEL domain and policy-level unit of analysis	Primary theoretical concepts used for abductive interpretation*	Recurring empirical patterns in reviewed IPS implementation studies	Policy-level analytic focus for cumulative knowledge-building
Policy legitimacy: political-administrative decision chains through which IPS is interpreted, authorized, mandated, or contested across health, employment, and welfare policy systems	Social citizenship and social rights [24]; deservingness criteria and conditional solidarity [25]; policy paradigms and definitions of legitimate goals [26]	Fit (or conflict) between IPS values and welfare/labor-market regimes: activation and conditionality, disability definitions, “readiness” logics and “vulnerability framing”; political commitment to recovery-oriented employment; advocacy and coalitions that legitimate IPS.	How political-administrative decisions and policy paradigms confer legitimacy on, authorize, contest, or marginalize IPS, and how conflicts between recovery-oriented, choice-based employment and conditionality, readiness, deservingness, or vulnerability logics are resolved, displaced, or reproduced
Policy embedding: multi-level and cross-sector implementation structures through which authority, resources, accountability, coordination, support, and discretion are organized and routinized beyond pilot implementation	Implementation structures as units of analysis [27]; complexity of joint action and multiple decision/clearance points [28], the integrated implementation model: from policy design to feedback from implementation outcomes [29], cross-sector coordination [30], and institutional logics and conflicting or coexisting sectoral logics [31]	Policy mandates may be explicit or rhetorical; decision and funding chains may be intact or break down across levels; stability/earmarking of funding; implementation infrastructure (training, learning collaboratives, fidelity review, shared performance data); cross-sector alignment via agreements, co-funding, co-location; local champions can buffer weak national coordination	How authority, resources, accountability, coordination, implementation support, and discretion are connected across administrative levels and sectors; and where mandates, funding arrangements, purchasing frameworks, or coordination mechanisms transmit IPS principles during sustainment and scale-up
Policy learning: structured implementation support, governance-level feedback loops, and adaptation through which implementation experience informs operational and policy decisions over time	Implementation outcomes and cumulative measurement [32], learning collaboratives and learning communities as multilevel dissemination, implementation-support, and feedback infrastructure [33, 34], dynamic sustainability and continuous intervention–context fit [15]; sustainability capacity [17] and documented adaptation of interventions and implementation strategies [16, 35]	Structured implementation-support capacity; learning communities associated with sustainment and scale-up; monitoring, evaluation, fidelity review, and performance data used to reinforce implementation quality and inform decisions; limited comparative measurement of sustainment, penetration, and reach; adaptation and fidelity challenges when IPS is extended to new populations, settings, or delivery systems	How structured implementation support, governance-level feedback loops, fidelity review, evaluation, implementation-outcome data, and adaptation are organized across levels and sectors, and how these learning structures inform sustainment, scale-up, and scale-out decisions over time

* Theoretical concepts are listed under the domain where they carried their primary analytic emphasis; several informed more than one domain.

### Policy legitimacy: institutional authorization and decision chains

Policy Legitimacy refers to whether IPS’s principles and objectives are regarded as normatively appropriate, institutionally compatible, and politically authorizable within a given welfare and labor-market regime. Its unit of analysis is the decision chain through which IPS is authorized, contested, or marginalized. Policy implementation research conceptualizes implementation as a multi-step chain of decisions linking high-level policy directives to frontline practice [28]. The unit of analysis is defined as a process that begins with ‘policy formulation’ and ‘policy design’ within legal and administrative frameworks and culminates in the explanation of variation in outputs [29]. Scaling EBPs may be regarded as an institutional logic competing for legitimization and institutional mandates with other policy logics: action may involve designing institutions as well as managing implementation [36]. In Hall’s terms, paradigms are shared frameworks of ideas and norms that define legitimate goals, instruments, and target populations [26]. The institutional logics perspective shifts attention from the policy legitimacy to congruence among policy design, intervention principles, organizational logics, and accountability mechanisms [31].

Labor-market participation among marginalized populations is a long-standing welfare-state question concerning how social citizenship, social rights, and responsibilities are defined [24]. IPS offers a distinctive evidence-based response to this question that aligns with “social investment” approaches that emphasize participation and capability-building as routes to inclusion [37]. However, in public policy, deservingness criteria vary across policy sectors, and public attitudes toward social security and the welfare state differ across electorates, whose preferences ultimately shape political agendas through electoral accountability [25]. Over recent decades, welfare-system reforms have more often followed a ‘workfarist’ than a social-investment orientation [38], despite evidence that stringent benefit-sanction systems can adversely affect vulnerable groups, including through financial hardship, housing instability, and worsened mental health [39, 40]. Pierson’s account of incremental welfare-state change suggests that reforms often become politically viable through recalibration of existing institutions rather than through overt welfare expansion or retrenchment [41]. Evidence-based practices such as IPS should therefore be understood as one reform agenda among others.

#### Decision-making authority

Across studies from the United States and Australia, IPS reportedly expanded when high-level policy intent was present: explicit political prioritization or high-level administrative decisions were linked to the model’s expansion [42, 43]. In Australia, broad dissemination increased visibility, but a significant policy shift resulted from direct ministerial-level engagement, supported by economic and implementation arguments [43]. In the United States, executive-level mandates have been described as enabling expansion by clarifying priorities and signaling that IPS is not optional [42]. In contrast, studies from Sweden and Finland suggest that the absence of clear political decisions and concrete operational follow-through is associated with fragile or short-lived programs [44, 45]. In studies of implementation infrastructure in the United States, where recovery-oriented employment was substantively prioritized—reflected in budgets, performance indicators, and service expectations—IPS penetration and sustainment tended to be stronger [33, 46]. In Sweden, administrative hesitancy to promote sustainment was attributed to weak decision-making authority [47].

#### Contested policy goals and entitlements

IPS became more policy-salient when aligned with broader participation- and inclusion-oriented policy framework [48, 49]. In Denmark, divergent institutional logics were described as a key source of tension when IPS was implemented within public employment services, with organizational priorities oriented to compliance and caseload processing rather than individualized services [50]. Studies from Finland and Denmark suggest that when employment services are key institutional counterparts, IPS may drift toward a peripheral position, perceived as an autonomous unit outside the organization’s core practice [45, 51]. Studies from Norway and the United States show that illustrate how IPS implementation and scale-out can depend on aligning employment-oriented policy goals with stakeholder understandings of the target group: framing people with mental illness primarily as vulnerable and at risk of being harmed by work could reinforce skepticism about employment as health-supportive, while scaling IPS out to new population groups required more intensive stakeholder engagement to ensure consistency of policy goals [52, 53].

#### Path-dependent policy constellations

Across the broader IPS implementation literature, IPS may remain marginal when recovery and employment are mainly rhetorical aspirations, or when welfare and service-delivery rules discourage vocational goals [22, 52, 54]. New Zealand and Norway illustrate two different forms of policy-level slippage. In Norway, national policy documents emphasize employment as a key objective of health policy. However, the national health authority separated employment specialist functions from healthcare services, reflecting a system-centered implementation approach [52, 55]. In New Zealand, despite policy reform, there was no requirement to modify services in line with evidence-based practice [54]. An Australian example shows that, without sustained policy enforcement, programs may start to drift and operations may revert to old ways of working [43].

### Policy embedding: institutionalized implementation structures

Policy Embedding refers to the formation and stabilization of an implementation structure: the cluster of organizational units, actors, mandates, resources, accountability mechanisms, and support routines through which IPS is delivered and maintained beyond the pilot implementation. Policy implementation research (PIR) explains variation in outputs by tracing how policy intent is translated through a multiorganizational implementation structure and enacted through professional routines and frontline discretion [29]. ‘Top-down’ PIR accounts emphasize reducing “slippage” by aligning incentives and responsibilities, and delegating implementation to agencies whose mandates align with the policy objectives [56]. For example, implementation has been conceptualized as a chain containing multiple decision and clearance points, at each of which the original policy purpose may be delayed, reinterpreted, weakened, or displaced [28]. “Bottom-up” accounts emphasize that policy processes are indeterministic and shaped by frontline discretion and fragmented jurisdictional, organizational, and collaboration arrangements that do not derive from the implementer’s regulatory authority [57, 58]. Hybrid implementation models have also been described, in which clear mandates and adequate resourcing create feasibility, while local ownership and problem-solving capacity enable adaptation and routinization [29]. The concept of the implementation structure provides a rationale for defining the relevant unit of analysis as the network of organizational actors assembled around a policy problem rather than any single formal organization [27].

#### Vertical embedding

In the United States, legislation mandated access to employment services for people with severe mental illness improved penetration and reach [59]. In the Netherlands and the United States, sustainment was supported by direct national funding schemes [60, 61]. In the Netherlands, the inclusion of IPS in national strategies and guidelines was reported to guide political and administrative decision-making at the lower levels [60]. Studies from the United States and Australia describe scale-up as vulnerable to “slippage” across a multi-step decision chain: high-level endorsement may not translate into clear mandates, stable or earmarked funding, or implementation-support infrastructure [42, 43]. In Finland, incoherent national-to-local policies were found to render sustainment dependent on agency leadership [45].

#### Horizontal embedding

Formal cross-agency coordination among responsible agencies was reported to have positive implications for penetration in the United States and Spain [61, 62] and for aggregate-level service quality in the United States [46]. Barriers arise when employment is not treated as a health-service goal, as shown in IPS implementation studies from the United States and Sweden, when employment services are organized around readiness or compliance logics [44, 63], or when data-sharing and referral responsibilities are unclear. The Nordic studies describe a typical response to weak cross-sector governance: implementers adapt IPS to fit existing systems, potentially compromising fidelity or limiting reach [52, 63]. In studies from the United States, facilitators of sustainment include formalized cross-sector arrangements and routine referral pathways [33, 46, 64].

### Policy learning: implementation evidence infrastructure

Policy Learning refers to sustained implementation evidence infrastructure through which implementation experience informs IPS practice and policy over time. The definition includes governance-level feedback loops in which implementation results inform system-level decision making and model refinement. Frameworks such as EPIS and PRISM highlight implementation-support and learning functions as important for sustainment, and they recognize the interaction between inner and outer contexts [7, 12]. The sustainability capacity perspective acknowledges the importance of system-level structures for sustainability [17]. However, these accounts less explicitly conceptualize policy-level learning structures as units of analysis. The capacity-building function is central to implementation-support models, in which implementation support practitioners build provider implementation capacity [65] and support systems strengthen delivery systems’ capacity for implementation [6]. The learning-collaborative model adapted by the originators of IPS may be more effective than low-intensity strategies for improving provider practices, although the evidence remains inconclusive [34, 66].

Policy implementation research adds that such learning structures are not only mechanisms for maintaining implementation quality but also a mechanism through which operational experience can recalibrate administrative and policy decisions. The integrated implementation model [29] links monitoring and evaluation back into subsequent policy design and implementation management through a feedback mechanism. The concept of implementation structures directs attention to the network of organizational actors assembled around learning functions [27]. Research on public-sector coordination adds that such learning depends on how information, accountability, and joint action are organized across administrative levels and sectors [30].

#### Structured implementation-support capacity

IPS studies from the United States show that dissemination benefited from structured support models, such as learning collaboratives that combine national- or state-level administrative leadership with regional- and local-level implementation support [34, 46]. In the United States, participation in the IPS learning community was associated with more durable programs and greater scale-up [67]. In implementation studies from the United States and Sweden, continuous evaluation was reported as a factor associated with sustainment [46, 47].

#### Governance-level feedback loops

Feedback-oriented evaluation was often organized by national, state, or regional organizations and outside experts, often alongside training and technical assistance. Evaluation and monitoring data were used to increase accountability, motivate decision-makers to increase funding, support national consensus-building, and inform local supervision based on achieved results [46, 61]. In some implementation contexts in the United States, achieving fidelity above a specified threshold was used as a requirement for funding [68], illustrating how fidelity review could serve as a feedback mechanism. In Australia, monitoring and evaluation were used to encourage leaders to reinstate high-fidelity services after implementation drift [43].

#### Transferability and scale-out

Scale-out studies indicate that fidelity is harder to maintain when IPS is extended to new populations or delivery systems, creating tensions between adhering to fidelity and adapting services to unmet needs. A United States IPS adaptation study found that maintaining fidelity was perceived challenging when scaling out to populations without a primary diagnosis of severe mental illness; administrators could nevertheless endorse adaptations to address gaps in employment services for clearly defined underserved populations, either by seeking to preserve fidelity standards without adaptation or by accepting lower fidelity as a trade-off for reaching additional groups [69]. In the Netherlands, stakeholders have found the IPS fidelity scale rigid when applied to new populations and delivery systems [49]. Studies from Nordic welfare-service contexts suggest that unplanned modifications or “hybrid” programs that dilute IPS’s core principles may emerge when competing institutional logics remain unresolved [44, 51, 63, 70].

## Discussion

This integrative review proposes the Legitimacy–Embedding–Learning (LEL) framework as a policy-level analytic framework for studying IPS sustainment and scale-up. The framework organizes recurring external-context patterns into three policy-level domains: Policy Legitimacy, Policy Embedding, and Policy Learning. Policy Legitimacy addresses policy authorization and decision chains linking high-level policy decisions to implementation; Policy Embedding addresses multi-level and cross-sector implementation structure; and Policy Learning refers to structured implementation support, governance-level feedback loops, and model refinement. By specifying these domains, LEL identifies policy-level objects around which cumulative and comparative evidence can be developed.

For implementation science scholars, the framework positions external context as a central analytical concern in sustainment and scale-up research. Existing frameworks acknowledge external context, implementation phases, adaptation, sustainability capacity, and intervention–context fit [12, 13, 15, 17]. However, empirical implementation research has often treated external context as a broad background condition rather than as a specific policy-level object of analysis [9–11]. LEL addresses this gap by conceptualizing external context not merely as external determinants but as a dynamic configuration of legitimacy, embedding, and learning. These analytical units may facilitate exchange between implementation science and policy implementation research by linking policy-process concepts to observable implementation outcomes.

Policy implementation research examines how politically or administratively formulated goals are translated into practice through implementation structures, routines, and discretion [28, 29, 57]. In EBPs, in contrast, key implementation outputs are predefined through model principles and research-backed assumptions about functionally important components [2]. This reframes implementation failure, slippage, and adaptation. For IPS, the reference point is therefore layered: political-administrative intent on the one hand and, on the other, recovery-oriented principles, outcome-predictive elements, and implementation supports. For welfare-state research, LEL suggests that evidence-based CPIs can be analyzed as sites of institutional recalibration and reconfiguration: the evidence-based practice implementation movement may be perceived as one logic competing or coexisting with other service-system logics for legitimacy, mandates, and organizational authority [31, 36]. Through its recovery-oriented principles, IPS is also an evidence-based approach to long-standing welfare-state questions about labor-market participation, social citizenship, social rights, and public-service responsibilities toward marginalized groups [24, 25, 38]. Comparative studies of EBPs such as IPS may respond to calls for stronger links between macro-level accounts of welfare-state change and micro-level empirical studies of policy implementation.

Housing First offers a plausible comparative case for comparing this work’s findings with findings on other CPIs. Like IPS, HF is a fidelity-based intervention for marginalized groups whose principles may challenge established service-system routines: studies report recurring tensions around the harm reduction approach and established practices [71, 72], show that implementation requires systemic rather than merely programmatic change [73], and suggest that legitimacy depends on policy paradigms rather than evidence alone. Embedding problems are also visible in HF research, where policy mandates help sustain existing programs and enable new ones [73, 74]. Implementation and de-implementation are shaped by interactions between model requirements, organizational structures, and established service-system routines [63], while fidelity drift has been described not simply as implementation failure but as an organizational response to structural scarcity and competing mandates [75]. Consistent with the Learning domain, HF studies highlight external implementation teams [76], ongoing fidelity monitoring to prevent drift [77], and feedback of evaluation findings to governments to secure program resources [78].

Sustainment may involve ongoing adaptation as interventions are integrated into evolving local contexts [15]. Also, when evidence-based practices are extended to new contexts, model-defining elements with demonstrated or hypothesized links to outcomes should be specified and protected during adaptation [79]. Both these cases may require specifying whether adaptation concerns the recovery-oriented principle set, the outcome predictive elements, or the organizational and contextual fidelity elements that support their implementation. For example, IPS studies repeatedly identify leadership as important for sustainment [64, 80–82], and leadership factors are included in the fidelity model. However, these elements correspond to implementation science constructs which are considered determinants of implementation [35, 83]. Because the evidence base on scale-out and adaptation remains limited [22], the relationships between core principles, predictive elements, and contextual supports are a developing field of inquiry.

Future research may use the LEL framework to orient analyses of sustainment and scale-up toward a more systematic account of policy-level conditions. Empirically, LEL can be used as a framework for comparing whether policy legitimization and decision chains, implementation structures, and policy-learning arrangements are present, aligned, and durable across jurisdictions and service systems. The framework could also support mutual learning between IPS and other complex psychosocial interventions, such as Housing First, whose implementation challenges reveal important similarities. Finally, future research should examine scale-out as a process in which recovery-oriented principles, outcome-predictive components, and organizational contextual supports jointly interact with external context in sustainment and scale-up.

### Limitations

This study has several limitations. It is theory-building and integrative rather than a full systematic review or meta-analysis. By spanning three broad theoretical traditions, the synthesis prioritizes conceptual integration and does not comprehensively represent any one tradition; the proposed framework’s domains should therefore be treated as integrative and exploratory. Moreover, the review did not systematically compare welfare-state models, national policy regimes, or implementation settings; country and jurisdictional examples were used to ground the interpretation rather than as objects of systematic comparison. The empirical literature was drawn entirely from high-income welfare-state contexts, and the framework requires separate empirical examination before being applied to low- and middle-income settings.

### Conclusion

This integrative review identified three policy-level domains for analyzing the sustainment and scale-up of the Individual Placement and Support model: Policy Legitimacy, Policy Embedding, and Policy Learning. LEL can be used as a framework for comparing policy legitimization and decision chains, implementation structure, and policy learning shape sustainment and scale-up. As an IPS-derived framework, its relevance for other complex evidence-based psychosocial interventions should be tested through comparative research.
